# A human kidney and liver organoid‐based multi‐organ‐on‐a‐chip model to study the therapeutic effects and biodistribution of mesenchymal stromal cell‐derived extracellular vesicles

**DOI:** 10.1002/jev2.12280

**Published:** 2022-11-16

**Authors:** Vivian V. T. Nguyen, Shicheng Ye, Vasiliki Gkouzioti, Monique E. van Wolferen, Fjodor Yousef Yengej, Dennis Melkert, Sofia Siti, Bart de Jong, Paul J. Besseling, Bart Spee, Luc J. W. van der Laan, Reyk Horland, Marianne C. Verhaar, Bas W. M. van Balkom

**Affiliations:** ^1^ Department of Nephrology and Hypertension UMC Utrecht Utrecht The Netherlands; ^2^ Department of Clinical Sciences Faculty of Veterinary Medicine Utrecht University Utrecht The Netherlands; ^3^ Hubrecht Institute Royal Netherlands Academy of Arts and Sciences (KNAW) Utrecht The Netherlands; ^4^ Dept of Surgery, Erasmus MC Transplant Institute University Medical Center Rotterdam Rotterdam The Netherlands; ^5^ TissUse GmbH Berlin Germany

**Keywords:** 3Rs, EV‐based therapeutics, micro‐physiological models, renal injury

## Abstract

Mesenchymal stromal cell (MSC)‐derived small extracellular vesicles (sEVs) show therapeutic potential in multiple disease models, including kidney injury. Clinical translation of sEVs requires further preclinical and regulatory developments, including elucidation of the biodistribution and mode of action (MoA). Biodistribution can be determined using labelled sEVs in animal models which come with ethical concerns, are time‐consuming and expensive, and may not well represent human physiology. We hypothesised that, based on developments in microfluidics and human organoid technology, in vitro multi‐organ‐on‐a‐chip (MOC) models allow us to study effects of sEVs in modelled human organs like kidney and liver in a semi‐systemic manner. Human kidney‐ and liver organoids combined by microfluidic channels maintained physiological functions, and a kidney injury model was established using hydrogenperoxide. MSC‐sEVs were isolated, and their size, density and potential contamination were analysed. These sEVs stimulated recovery of the renal epithelium after injury. Microscopic analysis shows increased accumulation of PKH67‐labelled sEVs not only in injured kidney cells, but also in the unharmed liver organoids, compared to healthy control conditions. In conclusion, this new MOC model recapitulates therapeutic efficacy and biodistribution of MSC‐sEVs as observed in animal models. Its human background allows for in‐depth analysis of the MoA and identification of potential side effects.

## 1 INTRODUCTION

Extracellular vesicles (EVs) have been identified as mediators of a newly discovered inter‐cellular communication system. EVs are essential signalling mediators in various (patho‐) physiological processes, through the transfer of bioactive molecules such as RNAs and proteins between cells (de Jong et al., 2014; Gamez‐Valero et al., 2019; Stoorvogel et al., 2002). Mesenchymal stromal cell (MSC)‐derived small EVs (sEVs) have been reported to promote therapeutic activities comparable to MSCs themselves, through paracrine effects. In animal studies, injected MSC‐sEVs facilitated tissue regeneration and immune modulation in a variety of diseases models including liver, heart, and kidney disease (Borger et al., 2017; Fan et al., 2020; Lai et al., 2010; Pachler et al., 2017). Since the successful treatment of a graft versus host disease patient in 2014 (Kordelas et al., 2014), beneficial effects of MSC‐derived sEVs have been reported for various diseases, including kidney disease in human small clinical studies (Nassar et al., 2016; Sengupta et al., 2020; Warnecke et al., 2021).

A regenerative medicine approach based on the application of MSC‐derived sEVs seems promising for kidney diseases. Preclinical work has demonstrated that the kidney can be protected, and recover from acute and chronic kidney injury by MSC‐derived EVs (Bruno et al., 2013; Eirin et al., 2017; Gatti et al., 2011).

Broad clinical translation of therapeutic EVs requires further preclinical and regulatory developments, including elucidation of the mode of action (MoA), the establishment of GMP‐grade sEV preparations, and formulation of safety‐ and release criteria (Reiner et al., 2017). To enable required therapeutic actions, it is crucial for sEVs to localise to the target site. As for many drugs, absorption and metabolism in the liver can limit effective delivery to the required site of action (Kang et al., 2021; König et al., 2013). In a mouse model for acute kidney injury (AKI), EVs have been shown to preferentially localise to the kidneys, the site of injury (Grange et al., 2014). A recent systematic review demonstrates that systemically applied EVs predominantly localise to the liver, followed by spleen, lungs and kidneys (Kang et al., 2021). Knowledge on biodistribution in healthy and disease conditions is crucial to assess MoA, safety and efficacy of sEVs. Studies on biodistribution are generally performed through the application of labelled EVs in animal models, currently most closely approaching the complexity of human (patho‐)physiology (Kang et al., 2021). However, the use of animal models comes with ethical concerns and is time‐consuming and expensive. Moreover, many human physiological processes are not adequately represented in animal models (Mak et al., 2014). Assessment of biodistribution, efficacy and safety without the use of animal experiments and against a human background could vastly accelerate therapy development (Marx et al., 2020; Roth, 2021).

Recent developments in microfluidics systems and human organoid biology provide the opportunity to develop in vitro chip‐based models to study the (patho‐)physiology of human organs (Bauer et al., 2017; Maschmeyer et al., 2015). These human organ‐on‐a‐chip models provide several important advantages, including high screening capacity, use of human cells and reduction of animal experiments. Combining multiple (organoid‐based) organ models in a single microfluidic circuit, thus multi‐organ‐on‐a‐chip (MOC) models, allow the analysis of systemic effects potential therapies, including therapeutic EVs. These include biodistribution, efficacy and potential undesired (off‐target) side effects. Furthermore, representative human MOC models allow relatively fast and inexpensive pre‐selection before moving onward to performing animal experiments (Marx et al., 2020; Roth, 2021).

Here we describe a human organoid‐based MOC model comprising kidney tubuloids, human adult stem cell‐derived organoid‐like structures representing the tubule segments of the nephron, (Schutgens et al., 2019) and liver organoids, representing a crucial organ for drug metabolism in general, and a major site for EV accumulation specifically (Huch et al., 2015; Schneeberger et al., 2019). These organ models are combined via a microcirculatory system to study the regenerative potential and organ distribution of MSC‐sEVs in a model for acute renal injury. We demonstrate that hepatic and renal functions are maintained in our micro‐physiological system. Using a model for sub‐lethal acute renal injury, we demonstrate that our human micro‐physiological system accurately recapitulates the efficacy and biodistribution of therapeutic EVs, as observed in animal models. The complete human background of our model allows further in‐depth analysis of the MoA and the identification of potential off‐target side effects in the liver. As such, this in vitro set‐up could contribute to a reduction in the use of laboratory animals, at the same time facilitating the simultaneous screening of multiple candidate EV preparations or other potential therapies, which can be extensively monitored by a variety of functional and molecular parameters. Together, this will strongly advance the basic knowledge and development of EV‐based regenerative medicine approaches for kidney diseases and beyond.

## 2 MATERIALS AND METHODS

### 2.1 Cell culture

All cell cultures were kept in the humidified atmosphere of 95% air and 5% CO_2_ at 37°C.

#### 2.1.1 Renal tubuloid culture

Kidney tubuloids from two independent donors were established and maintained as described (Schutgens et al., 2019). Briefly, cortical kidney tissue (healthy tissue from resected kidneys due to kidney cancer) was digested for 45 min using 1 mg/ml collagenase (Sigma) to obtain tubular fragments, which were resuspended in Basement Membrane Extract (BME, Type 2, Pathclear; R&D systems), seeded and cultured in kidney growth medium (GM): Advanced DMEM‐F12 containing 100 U/ml penicillin, 100 μg/ml streptomycin (ThermoFisher Scientific), 1 mM HEPES (ThermoFisher Scientific), 1 mM GlutaMax (ThermoFisher Scientific), 1 mM *N*‐acetyl‐L‐cysteine (Sigma), 1.5% (v/v) B27 (ThermoFisher Scientific), 1% (v/v) R‐Spondin 3 (U‐Protein Express BV), 50 ng/ml EGF (Peprotech), 100 ng/ml FGF‐10 (Peprotech), 10 μM Rho kinase inhibitor, Y‐27632 dihydrochloride (AbMole), 5 μM A83‐01 (Tocris), and 0.1 mg/ml Primocin (Invitrogen). After tubuloid structures had formed, the culture was propagated by mechanical dissociation of tubuloids using a p200 pipette, followed by resuspension in ice‐cold Advanced DMEM F‐12 and centrifugation at 300 × *g* to wash away BME, after which the fragments were resuspended in fresh BME for re‐seeding.

The use of human tissue for the generation of tubuloids was approved by the medical ethical committee of the UMC Utrecht and with written informed consent from patients.

#### 2.1.2 2D tubuloid culture and maturation

Tubuloid fragments were suspended in ice‐cold Advanced DMEM‐F12 and collected into a 15 ml conical tube. Next, the tubuloids were dissociated into single cells by incubating with TrypLE Express Enzyme (ThermoFisher Scientific) at 37°C. The tubuloids were pipetted up and down 15–20 times with a 200 μl pipette tip to assist their dissociation. After 30–45 min of incubation, the single cell suspension was centrifuged at 300 × *g* for 5 min. The supernatant was aspirated and single‐cells were resuspended in 1 ml of KGM. The number of cells was counted and 22,400 cells were seeded onto a 96‐well cell culture insert containing a polycarbonate membrane with 0.4 μm‐diameter pores (Millicell). After reaching confluency, 2D‐cultured tubuloids were differentiated towards proximal tubule lineage in kidney differentiation medium (DM; Advanced DMEM‐F12 containing 1 mM GlutaMax, 1 mM HEPES, 100 U/ml penicillin, 100 μg/ml streptomycin, supplemented with 5 μM A83‐01). The medium was refreshed every 2–3 days for 14 days.

#### 2.1.3 Human donor liver biopsies for organoid initiation

For the initiation of human liver organoids, liver biopsies (0.5–1 cm^3^) from two independent healthy liver grafts were obtained during liver transplantation procedures at the Erasmus MC. The use of liver biopsies for research purposes was approved by the medical ethics committee of the Erasmus University Medical Center (MEC‐2014‐060) and with patients informed consent of the transplant recipients. The biopsies were stored in University of Wisconsin solution on melting ice until used for organoid initiation as described (Huch et al., 2015). For this, cells from biopsy samples were dissociated by collagenase‐accutase digestion, washed with cold Advanced DMEM‐F12 and after centrifugation at 300–400 × *g*, pellets were resuspended Matrigel (Corning), and droplets of this suspension were pipetted in 48‐well plates at a density of 3000–10,000 cells/well. After solidification of the Matrigel, one ml of medium (Advanced DMEM‐F12 containing 1% (v/v) N2 and 1% (v/v) B27, 1.25 mM *N*‐acetyl‐L‐cysteine, 10 nM gastrin [Sigma], 50 ng/ml EGF [Peprotech], 100 ng/ml FGF10 [Peprotech], 1% (v/v) R‐Spondin 3 [U‐Protein Express BV], 10 mM nicotinamide [Sigma], 25 ng/ml HGF [Tocris], 5 μM A83.01 [Tocris], 10 μM forskolin [Tocris], and, during the first 3 days of culture, 25 ng/ml Noggin [Peprotech], 30% (v/v) home‐made Wnt conditioned medium and 10 μM Y27632 [Sigma]). After 14 days, the culture was established and organoids were propagated by mechanical dissociation into small fragments which were washed with cold Advanced DMEM‐F12 and resuspended in fresh Matrigel.

#### 2.1.4 Liver organoid culture

Liver organoids (Huch et al., 2015) were suspended in Matrigel and cultured in liver expansion medium (LEM; Advanced DMEM‐F12 containing 1% (v/v) GlutaMax, 1% (v/v) HEPES, 100 U/ml penicillin, 100 μg/ml streptomycin, 1% (v/v) primocin, 1% (v/v) N2 [ThermoFisher Scientific], 1% (v/v) B27, 1.25 mM *N*‐acetyl‐L‐cysteine, 10 nM Gastrin [Sigma], 200 ng/ml EGF, 1% (v/v) R‐spondin 3 (U‐Protein Express BV), 100 ng/ml FGF10, 10 mM nicotinamide [Sigma], 25 ng/ml HGF [Peprotech], 10 μM Y‐27632, and 0.5 μM A83‐01). Organoids were split once a week by mechanical dissociation. Briefly, Matrigel was re‐liquefied by adding ice‐cold Advanced DMEM‐F12 directly to Matrigel droplets, using a 10 ml glass pipette, and organoids were dissociated into fragments by shearing with 200 μl pipette. Then the smaller fragments were resuspended in fresh Matrigel droplets, and cultured as above. The medium was refreshed every second day.

#### 2.1.5 Hepatocyte maturation

Liver‐derived intrahepatic cholangiocyte organoids (Marsee et al., 2021) were primed for hepatocyte‐fate maturation in liver pre‐differentiation medium (pre‐LDM; LEM supplemented with 25 μg/ml BMP‐7) for 3 days and subsequently in liver differentiation medium (LDM) for 7 days. LDM was prepared based on LEM without R‐spondin‐3, FGF10, gastrin, nicotinamide, Y‐27632, whilst 25 ng/ml BMP‐7 (Peprotech), 10 nM DAPT (Selleckchem), 30 μM dexamethasone (Sigma), 100 ng/ml FGF‐19 (Peprotech) were introduced to LDM. The medium was changed every 2–3 days.

#### 2.1.6 MSC culture

Bone marrow‐derived mesenchymal stem cells (MSCs) derived from two independent production batches from anonymous donors who provided written consent were provided by the UMC Utrecht Gene and Cell Therapy facility and cultured in Dulbecco's Alpha MEM (ThermoFisher Scientific) supplemented with 10% (v/v) fetal calf serum (FCS; Biowest), 100 U/ml penicillin, 100 μg/ml streptomycin, 0.1 μg/ml primocin, 200 μM L‐ascorbic acid (Sigma), and 1 ng/ml basic Fibroblast Growth Factor (ThermoFisher Scientific) (Prins et al., 2009).

#### 2.1.7 MSC differentiation

To assess the differentiation potential of MSCs, cells were cultured in adipogenic (alpha MEM, 10% FCS (v/v), 0.52 mM 3‐Isobutyl‐1‐methylxanthine, 0.2 mM Indomethacin, 1.7 μM insulin and 1 μM dexamethasone), chondrogenic (DMEM high glucose, 10% FCS, 0.1 mM dexamethasone, 0.17 mM Vitamin‐C, 10 mM β‐glycerophosphate, 100 U/ml penicillin, and 100 μg/ml streptomycin) or osteogenic (DMEM low glucose, 10% FCS (v/v), 10 ng/ml TGF‐β1, 6.25 μg/ml transferrin, 50 μg/ml Vitamin‐C, 100 U/ml penicillin, and 100 μg/ml streptomycin) differentiation medium for 14 days. Differentiation towards adipocytes was assessed by Oil‐Red‐O staining. For this, cells were washed twice with PBS and fixed in 4% paraformaldehyde in PBS for 20 min at RT. Then, cells were incubated in 60% isopropanol for 5 min and subsequently stained by incubation in isopropanol containing 0.5% Oil red O (Sigma). After washing with 60% isopropanol, excess dye was removed by 2–5 washes with ddH_2_O, followed by a 1 min incubation with hematoxylin. After two washes with ddH_2_O cells were visualised. Chondrogenic differentiation was verified by Safranin‐O staining. After fixation in 4% paraformaldehyde in PBS for 20 min at RT, cells were washed twice with PBS and incubated in 0.1% Safranin‐O (Sigma) in ddH_2_O for 15 min at RT. After three washes with PBS, images were recorded. Osteogenic differentiation was assessed by Alizarin Red S staining. After fixation in 4% paraformaldehyde in PBS for 20 min at RT, cells were washed twice with PBS and incubated in 2% Alizarin Red S (Sigma) in ddH_2_O for 15–20 min. After washing the cells twice with ddH_2_O images were recorded.

#### 2.1.8 Flow cytometry

The presence of expression markers was assessed by flow cytometry. MSCs were harvested by trypsinisation and labelled with the following antibodies: anti‐CD105‐PE (Southern Biotech; 9811‐09), anti‐CD11b‐PE (BD bioscience; 555388), anti‐CD14‐PE/Cy7 (Biolegend; 301814), anti CD34‐PE (BD bioscience; 345802), anti‐CD45‐PE/Cy7 (BD bioscience; 557748), anti‐CD73‐PE (BD bioscience; 550257), and anti‐CD90‐PE/Cy7 (BD bioscience; 561558). As isotype controls for PE/Cy7 (BD bioscience; 557872) and PE (Beckman Coulter; PN‐AO7796) were used. Cells were labelled in the presence of FcR‐Blocking reagent (Miltenyi Biotec) for 30 minutes at 4°C after which cells were washed twice with FACS‐buffer (PBS, 5% FCS, 0.1% sodium azide). Flow‐cytometric analysis (≥10^4^ events acquired) was performed using a Becton Dickinson FACSCanto II as described (Besseling et al., 2021).

### 2.2 Functional assays

#### 2.2.1 Inulin‐FITC diffusion assay

The para‐cellular permeability of 2D‐cultured renal tubuloids was assessed by measuring the passive diffusional permeability of 4 kDa inulin. Basolateral to apical inulin‐FITC diffusion was performed by incubating cell culture inserts in Hank's Balanced Salt Solution (HBSS; Sigma)/10 mM HEPES (apical), and HBSS/10 mM HEPES containing 0.1 mg/ml inulin‐FITC (4 kDa; Sigma) at 37°C, 5% CO_2_. After 60 min, 100 μl aliquots were taken from both compartments to measure the fluorescence (excitation at 485 nm, emission at 538 nm) and assess relative passive diffusion. Culture inserts without cells were used as controls.

#### 2.2.2 CDFDA transport assay

To determine MRP2 transmembrane transport functionality of 2D‐cultured renal tubuloids, 5(6)‐Carboxy‐2′,7′‐dichlorofluorescein diacetate (CDFDA; Sigma) was used as a specific fluorescent substrate, and MK571 sodium salt hydrate (MK571; Sigma) was used as a competitive inhibitor for MRP2. Before beginning these transport assays, two important steps were carried out: (1) HBSS buffer was pre‐warmed. (2) The 2D‐cultured inserts were washed carefully with pre‐warmed HBSS buffer to remove all the existing media. After CDFDA and MK571 were dissolved in pre‐warmed HBSS buffer to acquire their working concentration, HBSS containing 10 μM CDFDA was added to the basolateral side of the renal tubuloids in the absence or presence of 20 μM MK571 in the apical and basolateral compartments. After 60 min at 37°C in the dark, 50 μl samples were taken from apical and basolateral compartments, and fluorescence measured at 485/538 nm. Next, MRP2‐specific transport was calculated as the difference in CDFDA absorbance between the non MK571‐inhibited and MK571‐inhibited values.

#### 2.2.3 Rhodamine 123 transport assay

To assess Multidrug Resistance Protein 1 transport activity, liver organoids were pre‐incubated with 10 μM of the specific inhibitor Verapamil (Sigma) or vehicle in HBSS for 30 min. Organoids were then treated with 100 μM Rhodamine 123 (Sigma) in LDM for 20 min. After washing with PBS, green fluorescence signal was imaged using an EVOS FL Cell Imaging System (EVOS FL Auto; Life Technologies).

#### 2.2.4 Trans‐epithelial electric resistance analysis

The barrier integrity of the cell‐layer can be assessed by trans‐epithelial electric resistance (TEER) measurement. TEER values were measured on 96‐well inserts with a semi‐permeable membrane using an EVOM^2^ equipped with an STX‐100 M electrode (World Precision Instruments) by placing electrodes into both the apical and basolateral compartment of inserts with the renal tubuloids, the electrodes were covered by the culture medium. For this, inserts were briefly taken from the chips and placed in a container for a single insert. TEER measurements were performed every 2–3 days after starting the maturation of renal tubuloids and at shorter intervals for injury‐recovery experiments. Semi‐permeable inserts without cells were used as negative controls.

### 2.3 Immunohistochemistry and immunofluorescence staining

#### 2.3.1 Kidney tubuloids

Membranes with renal tubuloids were washed with PBS and fixed with 4% paraformaldehyde (PFA) for 15 min at RT or 4°C o/n. Samples were washed three times in PBS, incubated in 50 mM NH_4_Cl quenching solution for 5 min at RT, and permeabilised and blocked for 40 min in PBS containing 0.1% Triton X‐100 and 0.5% BSA. Afterwards, samples were incubated with primary antibodies (ZO‐1; 1:500 or when combined ZO‐1; 1:2000 and Na^+^/K^+^‐ATPase; 1:500) in PBS at 4°C o/n.

#### 2.3.2 Liver organoids

Liver organoids were fixed directly inside the MOC system with 4% PFA for 15 min at RT, and washed twice with PBS. In the next steps, fixed‐liver organoids were removed from MOC system, then 4 μm paraffin sections were prepared. Subsequently, samples were heated at 62°C for 15 min and dewaxed by xylene, followed by rehydration in gradient ethanol concentrations from 100% to 70%, and 5 min in water. Then, paraffin sections were incubated in antigen retrieval solution (Dako) for 30 min at 98°C. After balancing to room temperature, sections were treated with 20 mM NH_4_Cl solution for 10 min to reduce background autofluorescence and blocked with 10% goat serum in permeabilisation buffer (Bright Diluent, ImmunoLogic) for 1 h to avoid non‐specific antibody binding. Afterwards, samples were incubated with primary antibodies (Table 1) in permeabilisation buffer at 4°C o/n.

**TABLE 1:** Antibodies used in this study

Antibody target	Usage	Dilution	Host	Manufacturer, Cat. No.
Immunofluorescence				
ZO‐1	Primary	1:500	rabbit	Invitrogen, 40–2200
Na^+^/K^+^‐ATPase	Primary	1:500	mouse	Abcam, ab7671
Acetylated tubulin	Primary	1:1000	mouse	Santa Cruz Biot., sc‐23950
Albumin	Primary	1:1000	rabbit	Invitrogen, MA5‐32531
Keratin 19	Primary	1:150	rabbit	Abcam, ab76539
P‐glycoprotein	Primary	1:200	mouse	Invitrogen, MA1‐26528
E‐cadherin	Primary	1:100	mouse	BD Bioscience, 610181
Argininosuccinate synthetase 1	Primary	1:150	rabbit	Invitrogen, PA5‐82740
Hepatocyte Nuclear Factor 4α	Primary	1:100	rabbit	Santa Cruz Biot., SC‐8987
Goat anti‐rabbit Alexa Fluor 488	Secondary	1:1000	goat	Invitrogen, A11029
Goat anti‐mouse Alexa Fluor 555	Secondary	1:1000	goat	Invitrogen, A21422
Mouse anti‐rabbit Alexa Fluor 488	Secondary	1:200	mouse	ThermoFisher, A‐11029
Mouse anti‐rabbit Alexa Fluor647	Secondary	1:200	mouse	ThermoFisher, A‐11034
Rabbit anti‐mouse Alexa Fluor488	Secondary	1:200	rabbit	ThermoFisher, A‐11004
Rabbit anti‐mouse Alexa Fluor647	Secondary	1:200	rabbit	ThermoFisher, A‐11036
Immunoblotting				
Flotillin‐1	Primary	1:500	rabbit	Santa Cruz Biot., sc‐25505
TOM20	Primary	1:1000	rabbit	Santa Cruz Biot., sc‐11415
GAPDH	Primary	1:10,000	rabbit	Cell Signaling Techn., 2118
Lamin A/C	Primary	1:2000	goat	Santa Cruz Biot., sc‐6214
ATP5A	Primary	1:1000	mouse	Abcam ab110273
Swine anti‐rabbit HRP	Secondary	1:2000	swine	DAKO, P039901
Rabbit anti‐goat HRP	Secondary	1:2,000	rabbit	DAKO, P044901
Rabbit anti‐mouse HRP	Secondary	1:2,000	rabbit	DAKO, P026002
Flow cytometry				
CD105‐PE	Primary	1:100	mouse	Southern Biotech, 9811‐09
CD11b‐PE	Primary	1:100	mouse	BD bioscience, 555388
CD14‐PE/Cy7	Primary	1:100	mouse	Biolegend, 301814
CD34‐PE	Primary	1:100	mouse	BD bioscience, 345802
CD45‐PE/Cy7	Primary	1:100	mouse	BD bioscience, 557748
CD73‐PE	Primary	1:100	mouse	BD bioscience, 550257
CD90‐PE/Cy7	Primary	1:100	mouse	BD bioscience, 561558
Isotype control PE/Cy7	Primary	1:100	mouse	BD bioscience, 557872
Isotype control PE	Primary	1:100	mouse	Beckman Coulter, PN‐AO7796

After 3 washes with PBS, samples were incubated with secondary antibodies (Table 1) in PBS (for tubuloids) or permeabilisation buffer (for liver organoids) for 45 min at RT and washed in PBS 3 times. Finally, nuclei were counterstained with 1 μM DAPI in PBS for 5 min at RT.

#### 2.3.3 Periodic acid‐Schiff (PAS) staining

Endogenous peroxidase activity was blocked by treating the paraffin samples in Dual Endogenous Enzyme Block (Dako) for 10 minutes. PAS staining was performed with DAB substrate, and hematoxylin (Dako) was used to counterstain the nuclei. Imaging was performed using a confocal microscopy (Leica TCS SP8 X; Leica Microsystems) with water‐immersion objectives ranging from 10× to 63×. Used antibodies, dilutions, and manufacturers are listed in Table 1.

### 2.4 Small extracellular vesicle isolation and labelling

#### 2.4.1 sEV isolation

sEVs were isolated from conditioned medium of MSCs by differential ultracentrifugation as previously described (van Balkom et al., 2013; Van Rhijn‐Brouwer et al., 2019). Cells were grown to 80% confluence in T175 flasks after which the culture medium was replaced for EV‐collection medium (MSC culture medium without FCS and Primocin). After 24 h, the conditioned medium was harvested and centrifuged for 15 min at 1500 × *g* to remove cellular debris, after which larger microvesicles were removed by centrifugation for 30 min at 10,000 × *g* using a Beckman XE90 ultracentrifuge and a SW32 Ti rotor (Beckman Instruments). sEVs were isolated by centrifugation for 60 min at 100,000 × *g*, and subsequently washed twice by re‐suspension in PBS and centrifugation for 60 min at 100,000 × g, all in a Beckmann XE90 ultracentrifuge and a SW32 Ti rotor, after which the final sEV pellet was collected by centrifugation at 100,000 × *g* using a Beckman XE90 centrifuge with a SW60 Ti rotor.

#### 2.4.2 sEV labelling

MSC derived sEVs were labelled with a green fluorescent dye PKH67 (Sigma). sEVs were resuspended in 180 μl PBS and 20 μL of Diluent C containing 1:25 diluted PKH67 was added. They were mixed continuously for 30 s by gentle pipetting and incubated at RT for 5 min. Then, 500 μl of 10% BSA in PBS was added to terminate the reaction. Next, serum‐free media was added until the total volume reached the 3.5 ml. The sEV suspension was placed on top of a 0.971 M sucrose (in PBS) layer and centrifuged for 2 h at 190.000 × *g* to pellet sEVs while retaining the unbound dye on top of the sucrose‐layer. Pelleted EVs were resuspended in PBS, centrifuged at 100,000 x *g* and subsequently resuspended in appropriate buffer or medium for further analysis. All centrifugation steps were performed using a Beckman XE90 centrifuge with a SW60 Ti rotor. As a negative control, the full procedure in the absence of sEVs was performed.

### 2.5 Extracellular vesicle characterisation

#### 2.5.1 Nanoparticle tracking analysis

Vesicle size was determined using a Nanosight NS500 (Malvern) using the following capture settings: Camera Level 13, a detection threshold of 5, and auto blur mode for the analysis. Five one‐minute videos were captured for each analysis and serial dilutions of (labelled) sEV preparations were used for quantitation.

#### 2.5.2 Sucrose density gradient analysis

sEV pellets were resuspended in 250 μl 2.5 M sucrose, 20 mM TRIS HCl pH 7.4 and floated in a SW60 tube for 16 h at 190,000 × *g* using a linear sucrose gradient (2.0–0.25 M sucrose, 20 mM Tris‐HCl, pH 7.4) in a Beckman XE90 centrifuge with SW60 Ti rotor. Gradient fractions (250 μl) were collected from the top and used for subsequent immunoblot analysis.

### 2.6 Immunoblotting

Detection of sEV proteins was performed by immunoblotting as previously described (van Balkom et al., 2015). For immunoblotting, sEV and cell samples were diluted 1:1 in Exosome Sample Buffer (5% SDS, 9 M urea, 10 mM EDTA, 120 mM Tris‐HCl, pH 6.8, 2.5% beta‐mercaptoethanol) and heated (95°C, 5 min). For cell samples, cells were scraped from the culture surface, resuspended in lysis buffer (1% SDS and 0.1% Triton X‐100 in PBS with protease inhibitors [cOmplete Mini, EDTA free, Roche] and incubated on ice for 30 min. Genomic DNA was sheared through a 27G needle four times. SDS‐PAGE, protein transfer and blocking were performed as described (van Balkom et al., 2015). Subsequently, membranes were incubated in either of the following antibodies: rabbit‐anti‐GAPDH (Cell Signaling), rabbit‐anti‐Flotillin‐1 (Santa Cruz Biotechnology), goat‐anti‐Lamin A/C (Santa Cruz Biotechnology), mouse‐anti‐ATP5A (Abcam), or rabbit‐anti‐Tom20 (Santa Cruz Biotechnology; Table 1).

As secondary antibodies 1:2000 diluted affinity‐purified swine‐anti‐rabbit, rabbit‐anti‐mouse, or donkey‐anti‐goat coupled to horseradish peroxidase (Dako) were used. Antigen‐antibody reactions were visualised with enhanced chemiluminescence according to manufacturer's guidelines (Chemiluminescent Peoxidase Substrate, Sigma) and imaged using a GelDoc XR+ system (Bio‐Rad).

### 2.7 Multi‐organ‐on‐a‐chip experiments

Liver organoids and 2D‐cultured renal tubuloids were (co‐)cultured in the TissUse HUMIMIC Chip2 microfluidic model (TissUse GmbH) (Bauer et al., 2017) in which one compartment was designed to hold a 96‐well insert (MOC‐Chip2‐96). Prior to starting the experiment, chips were rinsed and filled with PBS, and connected to HUMIMIC Starter, operating at 500 mbar pressure and 500 mbar vacuum with a frequency of 0.5 Hz, at 5% CO_2,_ 37°C, then PBS was switched to LEM the next day.

**FIGURE 1:**
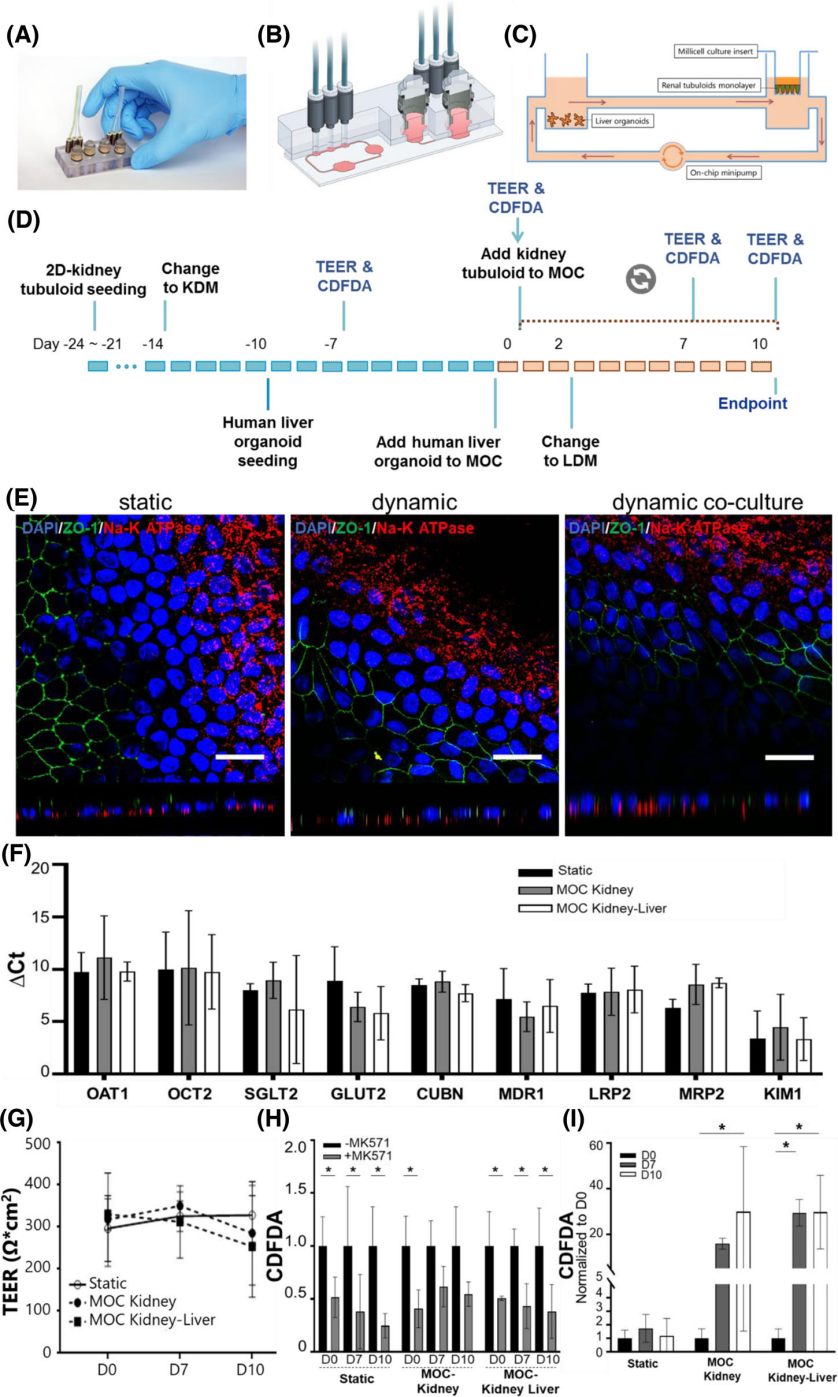
Characterization of 2D‐cultured renal tubuloids in static and dynamic culture. (A) Photograph, (B) cross‐sectional view and (C) schematic representation including organ representatives of the Chip2 organ chip used in this study. (D) Experimental timeline. KDM: kidney differentiation medium, LDM: liver differentiation medium (E). Immunofluorescence staining of 2D‐culture renal tubuloids in static, dynamic, and dynamic co‐culture (left, middle and right panel, respectively) with DAPI‐stained nuclei (blue), the tight junction protein ZO‐1 (green), and the Na^+^/K^+^‐ATPase (red) showing polarised epithelial cell layers in all conditions. (F) Relative mRNA expression of renal proximal tubule genes in 2D‐cultured renal tubuloids in static, dynamic, and dynamic co‐culture indicating expression of relevant proximal tubule genes (*n* = 3). (G) Relative trans‐epithelial CDFDA transport showing MRP2 function under each condition and (H) compared between different culture conditions (*n* > 3). *p < 0.05. Scale bar: 25 μm. (I) TEER measurements showing barrier integrity of renal tubuloids under different culture conditions (*n* > 3)

Ten days prior to combining liver organoids into MOC system, 22,400 single cells of liver organoids were cultured in 50 μl Matrigel as described above. At day 0 (D0) of the experiment, liver organoids in Matrigel were transferred to one compartment of the Chip2, and 2D‐cultured renal tubuloids in a Millicell culture insert were integrated into the second culture compartment. The circuits of Chip2 were filled with 750 μl fresh LEM supplemented with 25 μg/ml BMP‐7 for 2 days, and switched to LDM for the rest of the experiment, meanwhile the apical compartment of the renal culture insert still used kidney DM. The chip was then connected to HUMIMIC Starter, operating at 500 mbar pressure and 500 mbar vacuum at 0.5 Hz for the next 8 days. MOC‐Chip2 experiments were performed for 10 days. An amount of 50–150 μl medium per renal compartment and 250–500 μl medium per liver compartment were replaced every 2–3 days. The chips were monitored by functional assays and light‐ and confocal microscopy during experiments (Figure 1A).

### 2.8 Quantitative PCR analysis

For RNA isolation, we used either a general Trizol isolation method or the ISOLATE II RNA Mini Kit (250 cycles; Bioline). With the ISOLATE II RNA Mini Kit, we first added the lysis buffer to the samples for 3 min on ice cold (RLT buffer from the ISOLATE II RNA Mini Kit) after which the lysate was transferred to a new microcentrifuge tube and continued with the protocol of RNA isolation provided by the ISOLATE II RNA Mini Kit. Next, complementary DNA (cDNA) was synthesised using the iScript cDNA synthesis kit and a T100 Thermal cycler (Bio‐Rad) as described by manufacturer. Relative mRNA expression of the targeted genes was measured by real‐time qPCR using validated primers with SYBR Green Mixture (Bio‐rad) in a 96‐well format qPCR machine (CFX Real‐Time System; Bio‐Rad). Messenger RNA of the reference genes hP0 and GAPDH were used for normalisation of kidney tubuloids and liver organoids, respectively. All primers used in this study are listed in Table 2.

**TABLE 2:** Primers used in this study

Primer	Forward	Reverse
LPRP0 (P0)	CCATTCTATCATCAACGGG	TCAGCAAGTGGGAAGGTGT
SLC22A6 (OAT1)	TGTTCGGCTACCTTGCAGAC	CGTGTGTGAATGGGCATCCA
SLC22A2 (OCT2)	TGCATATTTTCGGCTTCCTC	ACCGGCTCACTAACATCTGG
SLC2A2 (GLUT2)	AATTGCTCCAACCGCTCTCA	CTAATAAGAATGCCCGTGACGAT
SLC5A2 (SGLT2)	TTCACCAAGATCTCAGTGGACAT	GAAGGTCTGTACCGTGTCCG
ABCB1 (MDR1)	TTGCTGCTTACATTCAGGTTTCA	AGCCTATCTCCTGTCGCATTA
LRP2	GTTCAGATGACGCGGATGAAA	TCACAGTCTTGATCTTGGTCACA
ABCC2 (MRP2)	CCCTGCTGTTCGATATACCAATC	TCGAGAGAATCCAGAATAGGGAC
HAVCR1 (KIM1)	TGGCAGATTCTGTAGCTGGTT	AGAGAACATGAGCCTCTATTCCA
CUBN	TTCTTACGGGGTCTGCTCAAA	GCAAGCCTTGGAATTTTCTCTCA
GAPDH	TGCACCACCAACTGCTTAGC	GGCATGGACTGTGGTCATGAG
ALB	GTTCGTTACACCAAGAAAGTACC	GACCACGGATAGATAGTCTTCTG
CYP3A4	TTTTGTCCTACCATAAGGGCTTT	CACAGGCTGTTGACCATCAT
CYP1A2	CCCAGAATGCCCTCAACA	CCACTGACACCACCACCTGAT
LGR5	GCAGTGTTCACCTTCCC	GGTCCACACTCCAATTCTG
HNF4α	GTACTCCTGCAGATTTAGCC	CTGTCCTCATAGCTTGACCT

### 2.9 Kidney injury model

Acute renal injury was mimicked at day 8 of the MOC experiment. Sub‐lethal injury to the 2D‐cultured tubuloids was induced by removing the culture inserts containing the renal cells from the MOCs and incubating them in kidney DM containing 2.5 mM hydrogen peroxide, which was added to the apical compartment only, for 1 h at 37°C, 5% CO_2_, followed by two washes with HBSS and TEER measurement. After this, inserts were transferred back to the MOC. MOCs containing injured renal tubuloids alone or in combination with liver organoids were maintained until day ten. TEER measurements and CDFDA transport assays were assessed at different time points after injury induction.

### 2.10 sEV treatment

After acute injury induced in renal tubuloids compartment, 500 μl of the circulating medium in the chips was replaced by 500 μL of kidney DM containing PKH67‐labelled sEVs isolated from approximately 10 million (1.01*10^7^ ± 7.21*10^5^) MSCs, corresponding to 2.12*10^9^ ± 2.65*10^8^ EVs. TEER measurement was performed on 2D‐cultured tubuloids after 1, 8, 24, 32, and 48 h of sEV exposure. EVs distribution was investigated by monitoring the PHK67 fluorescent dye by confocal microscope for liver and kidney compartments in the MOCs. After 48 h, at the end of the treatment, both culture compartments of the MOC were fixed by 4% PFA, and nuclei were stained by DAPI for further imaging processes as described.

### 2.11 Image analysis

To assess whether 2D‐cultured tubuloid cells alter their shape under the influence of basolateral flow, the ‘roundness’ of cells was determined based on the ZO‐1 staining pattern. In three separate images per condition, the ratio between the longest and shortest cell axis was determined for at least 10 cells, so that a perfectly round cell would have a ratio of 1. Similarly, the length of the primary cilium, stained for acetylated tubulin was assessed for at least 10 cells in five images per condition. ImageJ was used for image analysis and cilium length is normalised to the static culture condition.

To assess Keratin 19 expression, average intensity of the pixels staining positive was determined by dividing the total intensity of all stained pixels by the number of stained pixels using ImageJ.

sEV retention was assessed by normalising the PKH67 signal for the DAPI signal in at least ten images recorded after injury experiments and controls, both for the kidney and for the liver compartment, using ImageJ.

ImageJ was used to assess sizes of nuclei and nuclear fragments by measuring the surface of nuclei (>1000 nuclei per condition, representing three individual experiments).

### 2.12 Statistical analysis

Data were analyzed using a Student's *t*‐tests or two‐way ANOVA test using the Tukey HSD post hoc test where appropriate, and p‐values below 0.05 were considered significant.

## 3 RESULTS

### 3.1 Experimental setup

The MOC platform used during this study was designed by TissUse GmbH (Bauer et al., 2017), and consists of two separate circuits per chip. Each circuit is composed of two cell culture compartments interconnected by a micro‐channel system, allowing to co‐culture liver organoids and 2D kidney tubuloids in a 96‐well insert in separate culture spaces, connected under physiological flow conditions generated by an on‐chip minipump (Figure 1A–C). To generate a functional tubular epithelium separating a blood and a urine compartment, human renal tubuloids[24] are cultured on a semi‐permeable membrane, whereas the liver component consists of human liver organoids. The experimental timeline is illustrated in Figure 1D and further explained below.

### 3.2 Establishment and characterisation of the 2D‐cultured tubuloid‐based kidney component of the MOC model

To establish a functional 2D‐cultured kidney tubule monolayer on a semi‐permeable membrane that can provide a barrier between the blood‐ and urine compartments we used human renal tubuloids which have been established by our group (Gijzen et al., 2021; Schutgens et al., 2019). These tubuloids are cultured as 3D structures in droplets of BME and are physiologically and genetically stable during long‐term expansion. We physically and enzymatically dissociated the 3D tubuloid structure and seeded single tubuloid cells and grew these to a 2D layer on the semi‐permeable membrane of 96‐well culture inserts. Following a 14 day‐period of differentiation towards kidney tubule which has been published previously (Gijzen et al., 2021), the human kidney tubuloids formed a tight monolayer with morphology resembling that of a human renal proximal tubules (Supplemental Figure S1).

Polarisation of the kidney tubuloid monolayer grown under static conditions was shown by the basolateral expression of the Na^+^/K^+^‐ATPase and the apical honeycomb‐like localisation of the tight junction marker ZO‐1, both visualised through immunofluorescence staining (Figure 1E, left panel). When the renal cells were exposed to basolateral flow in the MOC system alone or in co‐culture with liver organoids, apical and basolateral expression of ZO‐1 and the Na^+^/K^+^‐ATPase respectively, were retained (Figure 1E, middle and right panel, respectively). Interestingly, the honeycomb‐like ZO‐1 staining appears stretched in the dynamic MOC conditions compared to the static culture conditions, suggesting that the renal cells adapt their morphology in response to flow. This is further substantiated by increased cilia length in the cells cultured under flow conditions as compared to statically cultured cells (Supplemental Figure S1).

The proximal tubule reabsorbs most of the glomerular filtrate and plays an important role in the uptake, metabolism and excretion of xenobiotics. Therefore, we next verified the gene expression level of several proximal tubule proteins by quantitative real time PCR. As illustrated in Figure 1F, organic anion transporter (OAT) 1 (*SLC22A6/A8*), organic cation transporter 2 (OCT2; *SLC22A2*), glucose transporter 2 (GLUT2; *SLC2A2*), sodium/glucose cotransporter 2 (SGLT2; *SLC5A2*), cubilin (*CUBN*), Multidrug Resistance Protein 1 (P‐glycoprotein; MDR1; *ABCB1*), LDL receptor related protein 2 (Megalin; *LRP2*), multidrug resistance‐associated protein 2 (MRP2; *ABCC2*), and kidney injury molecule 1 (KIM1; *HAVCR1*) were equally expressed at transcriptional levels at day 10 after the differentiation in static, dynamic, and co‐culture conditions.

To confirm transporter expression as functional proteins, we assessed trans‐epithelial transport of CDFDA, which is metabolised to the MRP2 substrate CDF, with or without MK571 as a selective inhibitor. MRP2‐mediated trans‐epithelial transport of CDFDA was clearly detected and is responsible for 40–60% of the total CDFDA transport, demonstrated by a strong and consistent inhibition of trans‐epithelial transport by MK571 (Figure 1G). Interestingly, although the relative contribution of MRP2 remains equal to static conditions, dynamic culturing conditions resulted in an approximate 25‐fold increase of absolute CDFDA transport at day10 (Figure 1H).

Functional 2D‐cultured renal tubuloids should provide a barrier between the blood‐ and urine compartments while retaining selective permeability for glucose, toxins and ions. To demonstrate barrier function, we monitored TEER, which gradually increased during the differentiation period, and inversely correlated to inulin‐FITC permeability (Supplemental Figure S1), indicating the formation of a leak‐tight epithelial barrier. In the next 10 days of the experiment in static and dynamic (co‐)culture, TEER values remained stable, even though there was a slight, non‐significant decrease in dynamic experiments regardless of the combination with liver organoids (Figure 1I). Taken together, 2D‐cultured renal tubuloids has generated a leak‐tight epithelial barrier after the differentiation and retained barrier function in static and dynamic culture conditions.

### 3.3 Establishment of the liver compartment

**FIGURE 2:**
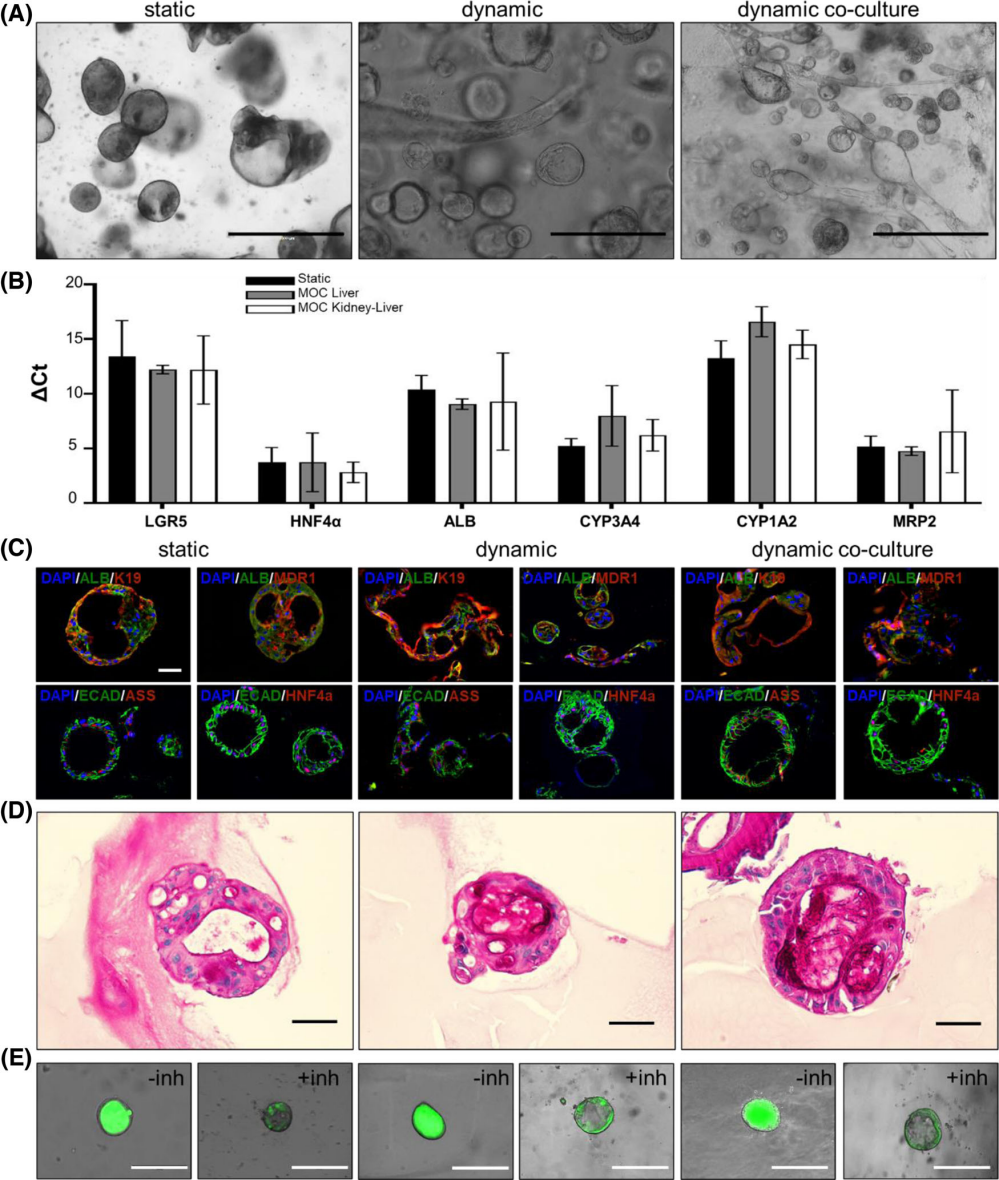
Characterization of liver organoids in static and dynamic (co‐)culture. (A) Liver organoids under static culture conditions show a spherical morphology, and form tubule structures under dynamic, and dynamic co‐culture conditions (left, middle and right panel, respectively; scale bar 1000 μm). (B) Relative expression of stem cell and liver marker genes (*n* = 3) and (C) proteins assessed by qPCR and confocal microscopy showing liver organoid maturation (scale bar 50 μm). (D) PAS staining showing glycogen storage capability of liver organoids under different culture conditions (scale bar 200 μm) and (E) MDR1‐mediated Rhodamine 123 transport in the absence (–inh) or presence (+inh) of the MDR1‐specific inhibitor Verapamil (scale bar 50 μm).

The liver component in our MOC model consists of 3D cultured human liver organoids (Schneeberger et al., 2019). Ten days before transfer to chips, single cells of liver organoids were seeded in a droplet of Matrigel and grown to spheroid‐like structures under static culture conditions (Figure 2A, left panel). Interestingly, after transfer to chips and exposure to flow, alone or in co‐culture with kidney tubuloids, liver organoids not only showed the circular‐like shape, but also obtained a tubular‐like shape (Figure 2A, middle and right panel).

In addition, we assessed the efficacy of differentiation into hepatocytes after 10 days. Compared to the morphology of undifferentiated liver organoids, differentiated organoids showed a denser, darker morphology and have a thicker cell layer (Supplemental Figure S2). qPCR analysis showed a downregulation of the stemness marker gene Leucine‐rich repeat‐containing G‐protein coupled receptor 5 (*LGR5*), a corresponding up‐regulation of the hepatic marker gene albumin (*ALB*) and constitutive expression of the hepatic marker genes Hepatocyte nuclear factor 4α (*HNF4A*), Cytochrome P450 3A4 (*CYP3A4*), Cytochrome P450 1A2 (*CYP1A2*), and MRP2 (*ABCC2*) after 10 days of differentiation (Supplemental Figure S2).

LRG5 expression remained down‐regulated when switched to dynamic culturing. HNF4α and CYP3A4 had higher expression in static conditions whereas ALB and MRP2 expression were slightly increased during dynamic (co‐)culture conditions (Figure 2B). To further confirm the differentiating hepatic phenotype of organoids, immunofluorescene analysis for ALB, keratin 19 (*KRT19*), Multidrug Resistance Protein 1 (MDR1; *ABCB1*), E‐cadherin (ECAD), argininosuccinate synthase 1 (*ASS*), HNF4α under all culture conditions was performed. Expression of cytoskeleton marker KRT19 and membrane marker ECAD indicated the differentiated organoids retained their polarised epithelial phenotype. Additionally, expression of hepatic markers ALB, MDR1, ASS, and HNF4α confirmed the differentiation towards the hepatocyte‐lineage. Interestingly, densitometric analysis of the cholangiocyte marker keratin 19 indicated a slightly increased expression in organoids cultured under dynamic conditions (Supplemental Figure S2), which, together with the formation of tube‐like structures under these conditions, suggests that dynamic culture conditions stimulate differentiation towards cholangiocytes.

An important function of liver is glycogen metabolism where glycogen is hydrolysed and broken down into glucose to immediately supply energy and balance the blood glucose levels during fasting state. Glycogen storage ability of liver organoids cultured under different conditions was assessed by Periodic acid‐Schiff staining, showing the accumulation of glycogen in differentiated organoid cells, in static and dynamic (co‐)culture conditions (Figure 2D, left, middle and right panel, respectively). Furthermore, we investigated MDR1‐mediated transmembrane transport activity of differentiated organoids under all culture conditions (Chen et al., 2018; Schneeberger et al., 2019). Organoids were exposed to rhodamine 123 (Rho123), a fluorescent MDR1 substrate, resulting in accumulation of fluorescence inside the lumen of organoids in all culture conditions (Figure 2E, –inh). In contrast, when the organoids were pre‐treated with Verapamil, the competitive inhibitor of MDR1, the fluorescent accumulation retained only in the cytoplasm of the cells, and no accumulation was found in the lumen of organoids, indicating the specific‐MDR1 transport for Rho123 (Figure 2E, +inh). Overall, our results demonstrate that hepatic function has been acquired by differentiated liver organoids in both static and dynamic cultivation.

### 3.4 Characterisation of MSCs and isolated sEVs

MSC‐derived EVs have been demonstrated to stimulate regeneration in a wide range of animal models for disease, including cardiac, cochlear and renal disease (Collino et al., 2015; Teng et al., 2015; Warnecke et al., 2021). To demonstrate the potential of our MOC model to assess the therapeutic action of MSC‐EVs, we developed a protocol to induce sub‐lethal kidney injury and assessed whether MSC‐derived EVs exhibit shows regenerative potential and biodistribution similar to those observed in in vivo models (Cao et al., 2020; Grange et al., 2014).

**FIGURE 3:**
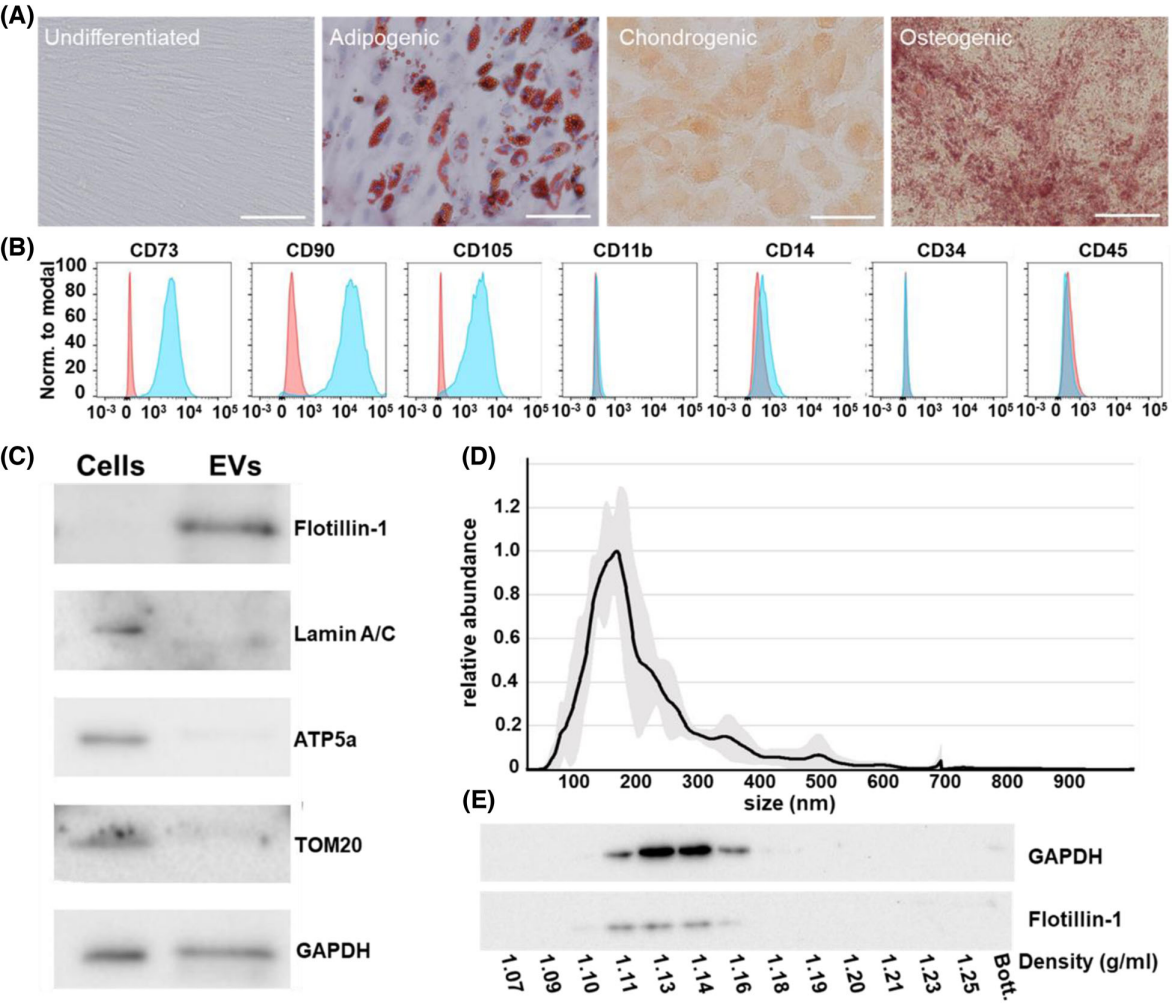
MSC and EV characterization. Cultured MSCs. (A) adhere to plastic and can be differentiated into adipogenic, chondrogenic and osteogenic lineages (scale bar 100 μm). They (B) express the essential surface markers CD73, CD90 and CD105 and lack CD11b, CD14, CD34 and CD45, all required to be absent from MSC, as assessed by flow cytometry (specific marker in blue, isotype control in red). Isolated sEVs are enriched in (C) Flotillin‐1 and depleted of the nuclear marker protein Lamin A/C and the mitochondrial proteins ATP5a and TOM20. GAPDH was used as a loading control. The (D) modal size of sEVs is 149 nm, as demonstrated by NTA, and (E) sucrose density gradient analysis followed by immunoblotting of fractions indicates that sEVs have a density of 1.13–1.14 g/ml (density of each fraction and bottom fraction [Bott.] indicated).

MSCs and EVs were characterised in accordance with the guidelines of the International Society for Cell & Gene Therapy (ISCT) and the International Society for Extracellular Vesicles (ISEV), respectively (Dominici et al., 2006; Lotvall et al., 2014; Théry et al., 2018; Witwer et al., 2019), and submitted to the EV‐TRACK knowledgebase (Van Deun et al., 2017). The first characteristic of MSCs, adherence to plastic, was used to select MSCs from bone marrow cells after Ficoll density gradient separation by plating 250,000 nucleated cells/cm^2^ in plastic flasks (Prins et al., 2009). The tri‐lineage differentiation capacity was verified by culturing MSCs in adipogenic, chondrogenic or osteogenic differentiation media and subsequent characterisation using Oil‐Red‐O, Safranin‐O staining, or Alazarin Red S staining, respectively, confirming that the MSC that were used as a source for EVs adhere to plastic and are capable of differentiation towards adipocytes, chondrobasts and osteoblasts (Figure 3A). The presence and absence of cell surface marker proteins were assessed by flow cytometry. As shown in Figure 3B, MSCs used as a source for sEVs express CD73, CD90 and CD105 on their cell surface, whereas, in accordance with the ISCT guidelines, CD11b, CD14, CD34 and CD45 cannot be detected.

Isolated sEVs were investigated for potential contamination by cellular debris through immunoblotting for the nuclear marker protein Lamin A/C and the mitochondrial marker proteins ATP synthase lipid‐binding protein (ATP5a) and Translocase of Outer Mitochondrial Membrane 20 (TOM20) (Figure 3C). Whereas these proteins are clearly detectable in MSC cell lysate, they are hardly detectable in sEV sample. In accordance, the sEV‐resident protein Flotillin‐1 can be identified clearly in the sEV sample. Glyceraldehyde‐3‐Phosphate Dehydrogenase (GAPDH) was used as a loading control and can be observed in both samples.

Nanoparticle tracking analysis was employed to assess EV size, demonstrating an average size of 345 nm and a modal size of 149 nm (Figure 3D). Additionally, sucrose density gradient analysis and subsequent immunoblotting for GAPDH and Flotillin‐1 reveal that the isolated sEVs have a density of 1.13–1.14 g/ml (Figure 3E). Both size and density are in the range of MSC‐derived sEVs as previously reported by us and others (Fafián‐Labora et al., 2020; Lai et al., 2012; Van Rhijn‐Brouwer et al., 2019). Taken together our data verify that we have successfully isolated sEVs from MSC cell culture supernatant (Witwer et al., 2019).

### 3.5 MSC‐sEVs reduce kidney injury in the MOC model

**FIGURE 4:**
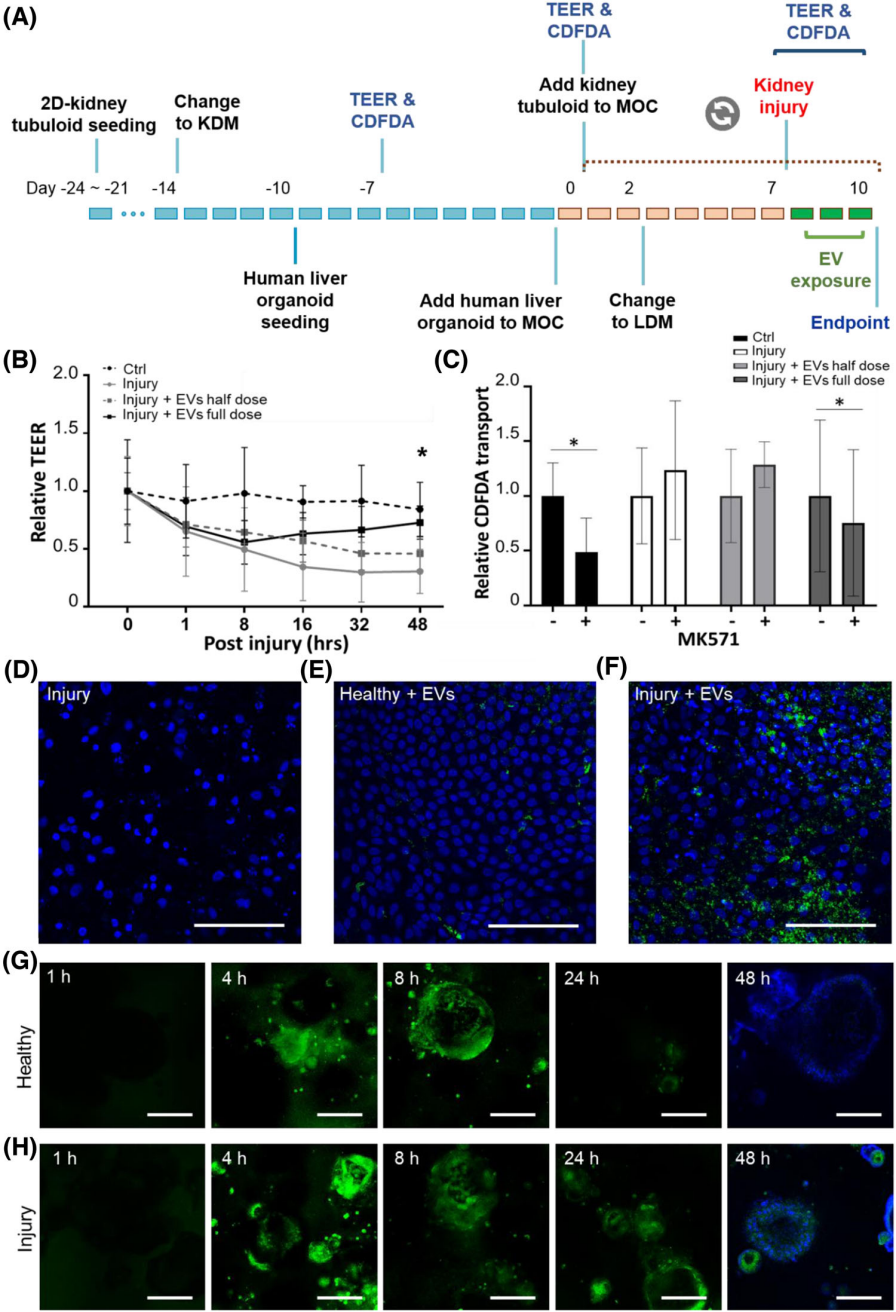
MSC‐derived sEV therapeutic effects and biodistribution. (A) The 2D‐cultured renal tubuloids were exposed to H_2_O_2_ after 7 days of dynamic co‐culture with liver organoids, followed by a 48 h sEV treatment. (B) TEER values remain stable in healthy controls, while a consistent decrease is observed after injury. Injured tubuloids treated with a full dose sEVs recover and show almost normalised TEER values (*p < 0.05 full dose vs. inury), whereas those that received the half dose show no significant recovery of barrier function (*n* = 3–6). (C) MRP2‐selective CDFDA transport after 48 h is lost in H_2_O_2_‐treated tubuloids and partially recovers in tubuloids treated with the full sEV dose, whereas no selective CDFDA transport can be observed in injured tubuloids exposed to half the sEV dose (*n* = 5–7). Injured tubuloids show (D) less and fragmented nuclei 48 h after injury induction, compared to (E) sEV‐treated healthy controls and (F) sEV‐treated injured tubuloids. Liver organoids accumulate sEVs in (G) healthy control and (H) injury conditions, with longer retention times in the latter condition. *p < 0.05; scale bar 100 μm

It is well‐established that reactive oxygen species (ROS), produced in response to for example ischemia/reperfusion (Kim et al., 2009) or cisplatin (Yoon & Kim, 2018) accumulation, can induce injury to renal tubular cells. in vitro, ROS‐induced cellular injury can be mimicked by hydrogen peroxide administration, and this is commonly done for renal tubular cells (Moon & Kim, 2019; Ye et al., 2015). Hence, to model acute kidney injury, renal tubuloids on cell culture inserts were exposed to H_2_O_2_ for 1 h to induce sub‐lethal cellular injury, and placed back into the chips. Next, sEVs from 10 million MSCs were applied to the circulating medium and barrier integrity and function were assessed monitored using TEER‐ and CDFDA transport assays (Figure 4A).

The renal epithelial barrier function was severely compromised directly after the 1 h exposure to H_2_O_2_, followed by a further decrease in TEER values during the first 24 h, after which they remained low until the end of the experiment (48 h). TEER values remained unaltered in the control MOCs containing untreated tubuloids, indicating we successfully modelled H_2_O_2_‐induced renal epithelial injury (Figure 4B). When MSC‐sEVs were administered to the circulating medium of H_2_O_2_‐exposed MOCs, the declining TEER values were inversed after 24 h and were significantly higher than injured tubuloids that received no EVs after 48 h. Injured tubuloids treated with a half sEV dose did not show significant recovery of the barrier function as assessed with TEER. MRP2 transport function after 48 h was also impaired after H_2_O_2_ exposure. After treatment with MSC‐sEVs the ability to selectively transport CDFDA consistently and significantly recovered, but did not reach the level of healthy controls due to variability between experiments. No functional recovery was observed for the tubuloids receiving no or a half dose of sEVs (Figure 4C).

MSC‐sEVs were fluorescently labelled with PKH67, a dye commonly used to monitor spatial distribution of sEVs in vitro and in vivo (Cao et al., 2020; van Balkom et al., 2013). Fluorescence microscopic analyses of the tubuloid monolayers shows the appearance of fragmented and debris of DAPI‐stained nuclei in injured renal tubuloids (Figure 4D). Analysis of DAPI stained nuclei and fragments shows no significant differences in size distribution nor average size could be observed between the three conditions. Interestingly, when EVs were administrated, the accumulation of sEVs on tubuloids in injury condition was 2.6‐fold (*p* < 0.001) higher compared to that in normal conditions (Figure 4E).

To explore further the potential therapeutic effects of MSC‐derived sEVs we assessed their distribution and recruitment to the liver compartment in our MOC model. As displayed in Figure 4F, MSC‐sEVs could be clearly detected in liver organoids at 4 and 8 h after application in MOCs that contained either control or injured renal tubuloids. After 24 and 48 h, however, sEVs retention in liver organoids that were in MOCs together with injured renal tubuloids was detectable at 2.1‐fold higher levels (*p* = 0.001) compared to the sEV signal in those combined with healthy control tubuloids (Figure 4G).

## 4 DISCUSSION

We here present a human microphysiological model for the assessment of therapeutic sEV efficacy and biodistribution. To our knowledge, we are the first to show an in vitro human organoid‐based circulatory model which demonstrates that after induction of kidney injury, MSC‐sEVs accumulate at the site of injury and reverse the impairment in kidney epithelial integrity and transport function. Furthermore we show that MSC‐sEVs also localise to the liver and that this is more pronounced after injury. Our model paves the way to accelerated development of sEV‐based therapeutics as it may allow for the elucidation of the MoA and potential safety and toxicity issues without the use of animal experiments and based against a human background.

Insights into systemic effects and molecular mechanisms of regenerative sEV therapy are absolute requirements before moving to a clinical phase, and experiments using animal disease models thus seem indispensable. Efficient pre‐screening to identify the optimal sEV preparation in the human situation before performing animal studies would dramatically impact the translational capacity and reduce costs and time required for animal experiments, and significantly address the three R's (**R**eplacement, **R**eduction and **R**efinement) for animal experiments.

### 4.1 Comparison to in vivo models

The MOC model presented here demonstrates that essential aspects regarding sEV efficacy and biodistribution, but also the effects of injury to a distant organ can be effectively mimicked in vitro and provides vast advances over standard in vitro assays. The regenerative effects of systemically‐applied MSC‐derived sEVs in our MOC model for acute kidney injury recapitulate earlier in vivo experiments in which such vesicles were intravenously injected in mice subjected to glycerol‐, cisplatin‐ or ischemia‐reperfusion‐induced AKI (Bruno et al., 2012; Cao et al., 2020; Collino et al., 2015). Interestingly, besides mimicking the regenerative effect of MSC‐sEVs in kidney injury models, our results on the differential distribution of sEVs, indicating preferred homing to injured tissue compared to control, are in line with the observations by Grange et al. and Cao et al., both demonstrating that a regenerative effect of MSC‐derived sEVs is accompanied by more intense and prolonged signal from labelled sEVs in diseased kidneys (Cao et al., 2020; Grange et al., 2014).

Furthermore, as anticipated from the data on EV biodistribution (Kang et al., 2021), we observe an initial homing to the liver, which gradually reduces over time, in our control experiments. Importantly, in our injury model, homing and/or retention of sEVs to the liver compartment is prolonged after H_2_O_2_ treatment of *only* the kidney cells. This observation is in line with the findings that sEVs appear more abundant in the liver of MSC‐sEV‐treated AKI mice, compared to their healthy counterparts as observed in two studies in which the biodistribution of MSC‐sEVs applied after kidney injury was monitored (Cao et al., 2020; Grange et al., 2014). Although it is suggested this is due to non‐specific accumulation of vesicles in excretory organs, another explanation could be that systemic effects of AKI injure the liver and induce an inflammatory response, which may create an environment prone to the adherence or absorption of MSC‐sEVs (Golab et al., 2009).

It is important to realise that mimicking this systemic, or inter‐organ communication requires a continuous circulation of the cell culture medium, carrying secreted factors from organ to organ, in a closed fashion. Thus, compared to an earlier study investigating the biodistribution of cancer EVs using an MOC setup in which liver‐ and kidney‐tissue slices were combined in parallel culture chambers (Tian et al., 2020), our organoid‐based MOC with continuous pulsatile medium circulation may more closely reflect human physiology. The closed circulatory circuit of our model provides advantages over individual statically‐cultured organoid culture. The shear stress induced by the pulsatile flow is sensed by both kidney and liver organoids, leading to adaptations of morphology, cell differentiation and function. In the 2D‐cultured renal tubuloids, cell alignment and increased cilia length are direct consequences of laminar shear stress. Additionally, the function of MRP2 is stimulated approximately 25‐fold, in line with the effects of flow observed in conditionally immortalised proximal tubule epithelial cells (Vriend et al., 2020). Furthermore, liver organoids form tube‐like structures and show increased keratin 19 expressions when exposed to flow, indicating (partial) differentiation of hepatocytes or remaining hepatic progenitor cells towards cholangiocytes. This observation underscores the true bi‐potential feature of the liver organoids as has previously been reported (Chen et al., 2018).

### 4.2 Organ‐organ interaction

The continuous circulation in our MOC model has the potential to truly model systemic organ‐organ interactions. This becomes evident from our observations that upon injury to the renal compartment, which is applied with the cell culture insert out of the chip, sEVs show longer retention in the liver organoids. This implies that injury to the kidney alone renders the liver compartment more prone to accumulate sEVs, similar to the observations in mouse models of AKI by Cao et al. (2020) and Grange et al. (2014). The interplay between the kidney and liver in human (patho‐)physiology is well known, illustrated by the occurrence of acute kidney injury in cirrhosis patients (Wang et al., 2021) and, vice versa, injury to remote organs, including the liver, in acute kidney injury (Grams & Rabb, 2012; Husain‐Syed et al., 2020). The systemic nature of this interplay has been investigated using small and large animal models (Gardner et al., 2016; Hakimizadeh et al., 2020). We show that also in our MOC model, at least in part, the interaction between the kidney and liver can be mimicked in vitro.

### 4.3 Limitations

Human MOC models like the model presented here offer great opportunities to assess therapeutic efficacy and potential side effects in a human background. In our model, the two most relevant organs to assess a MSC‐sEV‐based therapeutic approach for kidney injury were combined, providing proof‐of‐concept. Nevertheless, our model has limitations when it comes to mimicking an entire organism. It offers a solid starting point to extend the number of represented organs, as a more systemic representation of interacting human organs would of course offer broader insights into biodistribution and potential side effects. A combination with models for lungs would be highly relevant as these organs are known to accumulate EVs (Kang et al., 2021). With organ‐on‐a‐chip models available for this organ (Gkatzis et al., 2018; Schimek et al., 2020), this would be a relatively straightforward next step in our research. Furthermore, our model offers the possibility to add immune cells. Although MSC‐sEVs appear to be applicable in an allogeneic fashion as demonstrated in ageing mice (Dorronsoro et al., 2021) or mice with heart (Arslan et al., 2013) or kidney disease (Zhang et al., 2020), the incorporation of immune system components would be very informative regarding potential (adverse) immunological responses. Apart from the scientific and biological limitations, limited access to the MOC technology may hamper the applicability of this technology. However, this research field is rapidly expanding (Ingber, 2022), and MOC platforms are becoming available more and more.

## 5 CONCLUSIONS AND FUTURE PERSPECTIVES

Collectively, these data indicate that it is possible to combine 2D‐cultured renal tubuloids with liver organoids to study clinically relevant kidney‐liver interaction in dynamic MOC culture. Importantly, sEVs derived from human MSCs have shown to accelerate the recovery of renal tubuloids from oxidative damage by promoting the restoration of barrier integrity and recovery of functional transport. In addition, labelled MSC‐derived sEVs were detected to localise within liver and renal tubuloids in both healthy and injury conditions. Interestingly, kidney injury seemed to influence the accumulation of sEVs in the liver, illustrating organ‐organ interaction.

Our MOC model recapitulates findings on biodistribution and therapeutic efficacy of MSC‐sEVs as observed in animal models. Its human background allows for in‐depth analysis of the MoA and identification of potential side effects. These MOC models allow for further in‐depth research on the MoA on a human background, for therapeutic EV and pharmaceuticals. Our MOC model provides a basis for the development of more complex models, combining additional relevant organ models as a platform for accelerated development of therapeutic EVs.

## CONFLICT OF INTEREST

R.H. is CEO of TissUse GmbH, which produces and markets microphysiological systems. None of the other authors have any conflict to declare.

## Supporting information

Supporting Information

## Data Availability

The data that support the findings of this study are available from the corresponding author upon reasonable request.
