# Supplementary material for: A human kidney and liver organoid‐based multi‐organ‐on‐a‐chip model to study the therapeutic effects and biodistribution of mesenchymal stromal cell‐derived extracellular vesicles
Source: J Extracell Vesicles. 2022 Nov 16;11(11):12280. doi: 10.1002/jev2.12280 (PMC9667402; doi:10.1002/jev2.12280)
Supplement: Supplementary file 1 — Supporting Information [file JEV2-11-12280-s001.pdf]

Supplemental information

A human kidney and liver organoid-based multi-organ-on-a-chip model to study the therapeutic effects and biodistribution of mesenchymal stromal cell-derived extracellular vesicles.

Vivian V.T. Nguyen[1], Shicheng Ye[2], Vasiliki Gkouzioti[1], Monique E. van Wolferen[2], Fjodor Yousef Jengej[1], Dennis Melkert[1], Sofia Siti[1], Bart de Jong[1], Paul J. Besseling[1], Bart Spee[2], Luc J.W. van der Laan[3], Reyk Horland[4], Marianne C. Verhaar[1] and Bas W.M. van Balkom[1,5]

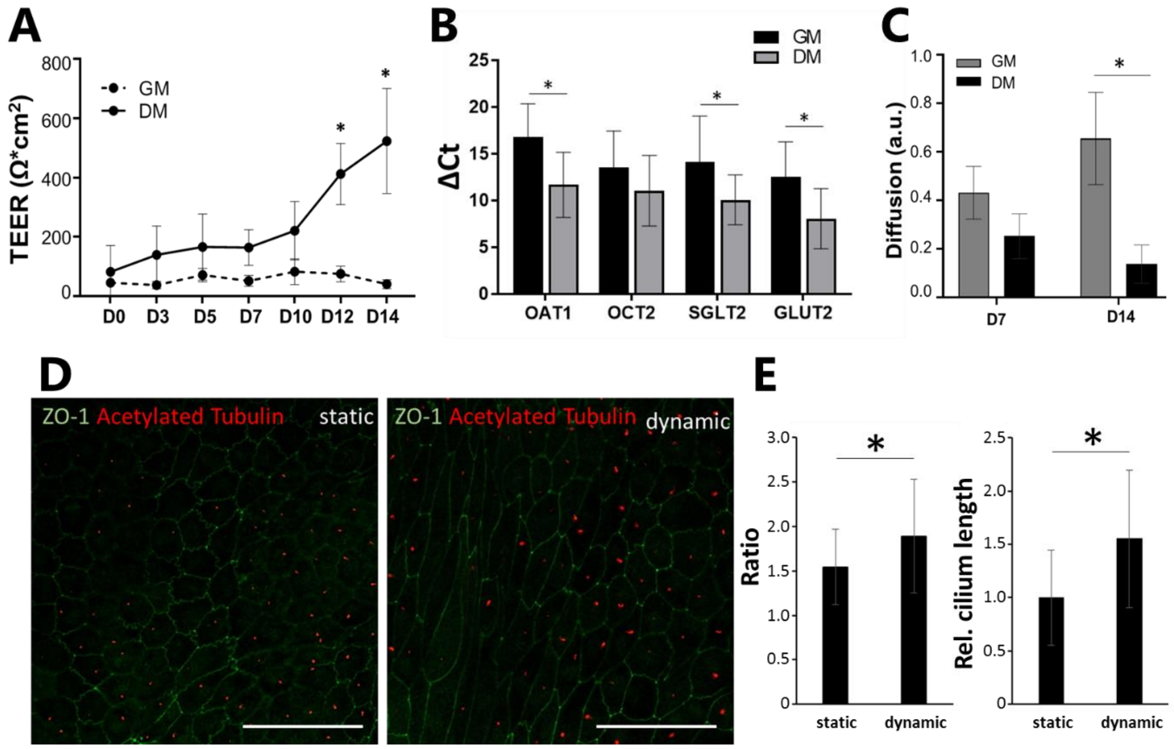

Supplemental figure S1: 2D-cultured renal tubuloids form a tight monolayer during differentiation and adapt to dynamic conditions. Assessment of (A) trans-epithelial electrical resistance (TEER) during the differentiation period shows a gradual increase of barrier function in cells in differentiation medium (DM) compared to those in growth medium (GM), accompanied by (B) increased expression of proximal tubule genes OAT1/3, SGLT2 and GLUT2. Passive diffusion of inulin-FITC (C) gradually reduces and is significantly lower when 2D-tubuloids are grown in DM compared to GM (indicated). Confocal images (D) of ZO-1 (tight junctions, green) and acetylated tubulin (cilia, red) stained 2D-cultured renal tubuloids maintained under static and dynamic conditions (indicated) were used to assess cell alignment by (E) assessing cell elongation (maximum cell diameter divided by minimum cell diameter) and (F) analysis of cilia length. *: $p < 0.05$; scale bar 50 µm;

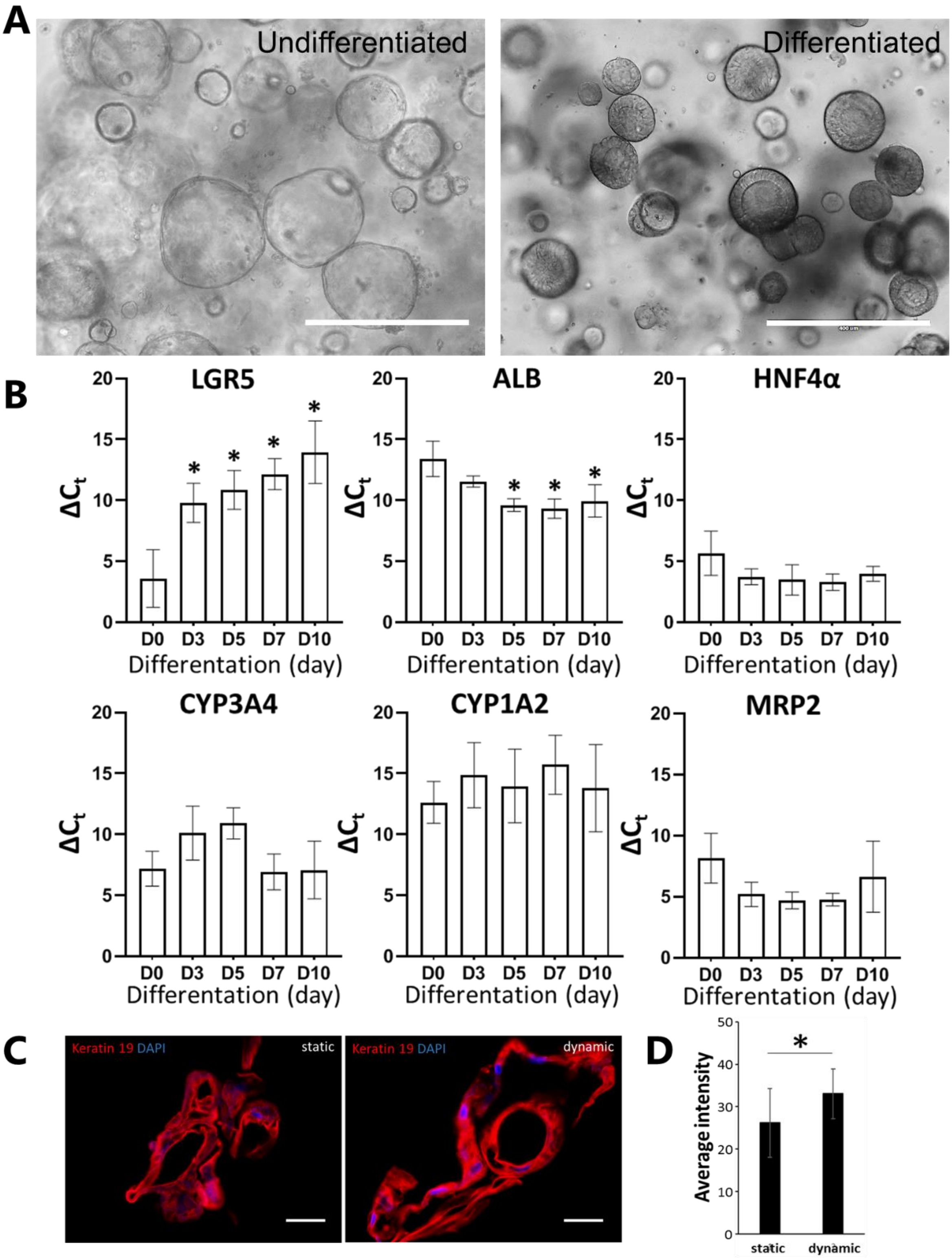

Supplemental figure S2: Differentiation of liver organoids. After ten days of differentiation, liver organoids obtain (A) a more dense phenotype as observed by light microscopy (bar = 400 µm) and (B) show a reduced expression of the stem cell marker LRG5. The liver marker gene albumin (ALB) is up-regulated while other genes remain unaltered (n=3-4). Quantification of (C) Keratin 19 (K19) staining reveals a (D) small but significant higher expression in liver organoids grown under dynamic (n=12) compared to static (n=7) conditions (*: $p < 0.05$; scale bar 25 µm).
